# From the European indoor radon map towards an atlas of natural radiation

**DOI:** 10.1093/rpd/ncu244

**Published:** 2014-07-25

**Authors:** T. Tollefsen, G. Cinelli, P. Bossew, V. Gruber, M. De Cort

**Affiliations:** 1 European Commission, DG Joint Research Centre, Institute for Transuranium Elements, Via E Fermi 2749, Ispra I-21027, Italy; 2 German Federal Office for Radiation Protection, Köpenicker Allee 120-130, D-10318 Berlin, Germany; 3 Austrian Agency for Health and Food Safety, Wieningerstrasse 8, Linz A-4020, Austria

## Abstract

In 2006, the Joint Research Centre of the European Commission launched a project to map radon at the European level, as part of a planned European Atlas of Natural Radiation. It started with a map of indoor radon concentrations. As of May 2014, this map includes data from 24 countries, covering a fair part of Europe. Next, a European map of geogenic radon, intended to show ‘what earth delivers’ in terms of radon potential (RP), was started in 2008. A first trial map has been created, and a database was established to collect all available data relevant to the RP. The Atlas should eventually display the geographical distribution of physical quantities related to natural radiation. In addition to radon, it will comprise maps of quantities such as cosmic rays and terrestrial gamma radiation. In this paper, the authors present the current state of the radon maps and the Atlas.

## INTRODUCTION

After the European Commission published the ‘Atlas of Caesium Deposition on Europe after the Chernobyl Accident’^(1)^, the Radioactivity Environmental Monitoring group of the Joint Research Centre (JRC) of the European Commission decided to embark on a European Atlas of Natural Radiation. This is in line with its mission, based on the Euratom Treaty, which is to collect, validate and report information on radioactivity levels in the environment.

This Atlas is intended to familiarise the public with the radioactive environment, to give a more balanced view of the annual dose that it may receive from natural radioactivity and to provide reference material and generate harmonised data for the scientific community. The Atlas should display the geographical distribution of certain physical quantities related to sources of natural radiation at different stages in the chain of physical causality. Eventually, the Atlas should comprise a collection of maps covering radon-related quantities. In addition, maps of other sources of exposure to natural radiation are planned, such as cosmic rays and terrestrial gamma radiation^(2)^.

## THE EUROPEAN INDOOR RADON MAP

Indoor radon yields, in most cases, the most important contributor to population dose. Most researchers consider its radioactive progenies to be the leading cause of lung cancer, second only to smoking^(3)^. Even relatively low exposure, corresponding to indoor radon concentration of 100 Bq m^−3^, significantly enhances the risk of lung cancer. Given the relevance to public health, some European countries have implemented a number of regulations to identify radon-prone areas where prevention and mitigation are considered particularly important.

When the JRC published an overview of radon surveys in 2005^(4)^, it showed that no two countries had used the same approach, in terms of survey design, measurement techniques and mapping strategies. With such differences in the choice of mapped quantity and in type of visualisation, the resulting maps were heterogeneous and incompatible across borders. As a consequence, collecting information on radon data from different countries and integrating them into a common framework implies a number of conceptual and technical challenges.

As the survey showed, indoor radon measurements are available from most European countries. At the 8th International Workshop on the Geological Aspects of Radon Risk Mapping held in Prague in 2006, the countries agreed to participate to a European Indoor Radon Map (EIRM). The mapped quantity intends to show ‘means over 10×10 km grid cells of (mean) annual indoor radon concentration in ground-floor rooms of dwellings’. This grid has been defined by the JRC and uses a projection (GISCO-Lambert azimuthal equal area) suitable at the continental scale. In order to respect data privacy, the participants, all of which are national competent authorities, aggregate their original data into the grid and calculate a set of statistics per cell: the arithmetic mean (AM), standard deviation (SD), AM and SD of ln-transformed data, minimum, median and maximum, as well as the number of measurements per cell. These statistics should be calculated using annual, averaged measurements made on ground floor of residential houses. Note that this procedure guarantees data protection, since the original data and their exact locations remain at the national level.

As these statistics arrive from the participants, the JRC carries out exploratory analysis, identifies errors and outliers and produces a diagnostic file for each country. Following that, the JRC integrates them into the European map and publishes updated versions at convenient intervals. For further details and preliminary results, given elsewhere^(5–7)^.

Choosing annual mean radon concentration on ground-floor rooms as the variable to be mapped has certain restrictions. In some instances, data providers have to estimate this quantity. If measurements are made over less than a year, usually some modelling with seasonal correction has to be applied. Nevertheless, this approach was adopted because most data are available for this variable.

Moreover, as many data providers also have remarked, most people do not live on ground floor (especially in cities with high population density) but on higher floors with generally lower radon exposure. In order to map exposure, either data must come from a carefully designed survey that reflects demographic reality (samples representative for population density and house occupation characteristics) or model-based correction to account for demographic representativeness must be performed. Since few national surveys are designed that way (see below) and, on the other hand, the demographic data are not available to the authors, they could neither choose the ‘design-based’ nor ‘model-based’ approach for creating a European radon exposure map, so this must be left to future efforts.

As of May 2014, 24 European countries participate to the map. It has >21 000 non-empty cells filled with data, stemming from >800 000 individual measurements in total, covering a fair part of Europe.

**Figure 1.**
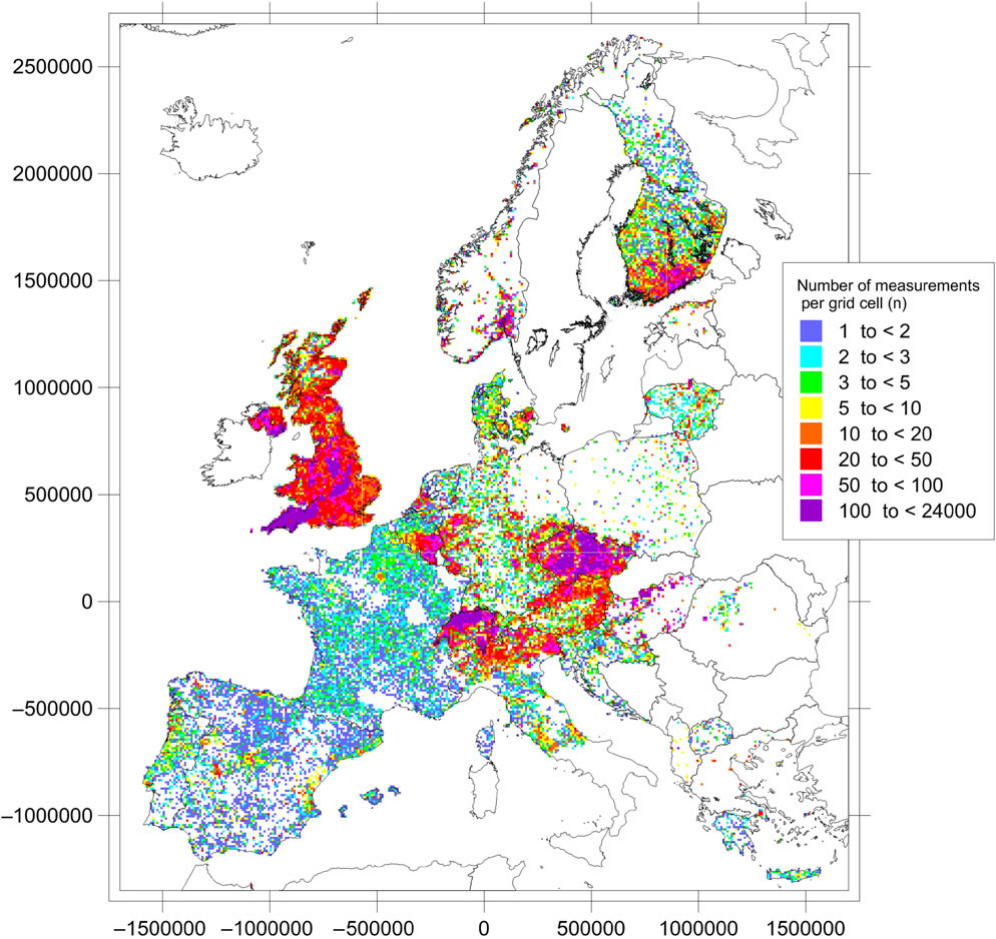
Number of measurements per 10 × 10 km grid cell. Data available until May 2014 included.

The number of measurements per cell and coverage of territory vary widely between the participating countries and between regions within those countries, as can be seen from Figure 1. Several countries have nominally covered >100 % of their territory with data, due to a border effect. At the other end of the scale, a few countries have sampled (or sent data for) <20 % of their territory. Thus, the map indicates the status of national surveys of indoor radon in Europe.

The wide ranges in sampling density depend on the design of the survey from which the data originate. In order to understand better the differences and their possible influence on the result, since 2010, the JRC has sent a quality-assurance questionnaire to the data providers, asking them about survey design, measurement methods, detectors used and data-processing techniques.

Results show^(8)^ that some countries have opted for homogeneous coverage of territory, whereas others (e.g. Austria) for a population-weighted estimate of radon concentration, which results in sampling density essentially proportional to population density. Five of the countries (Germany, the Czech Republic, Switzerland, Spain and Finland) have carried out more detailed surveys in high-radon areas. Finally, some datasets (Estonia, Greece, Romania and Poland) are mainly based on surveys in radon-prone areas.

As seen from Figure 1, many areas of Europe are still not covered by this map. Although a few uninhabited areas remain, reasons are missing data, because radon surveys are still ongoing, or national surveys have concentrated on high-radon areas. Yet, some countries (e.g. Ireland) chose survey designs, which produced datasets incompatible with the European mapping project, and others (e.g. Sweden) do not have a national database.

**Figure 2.**
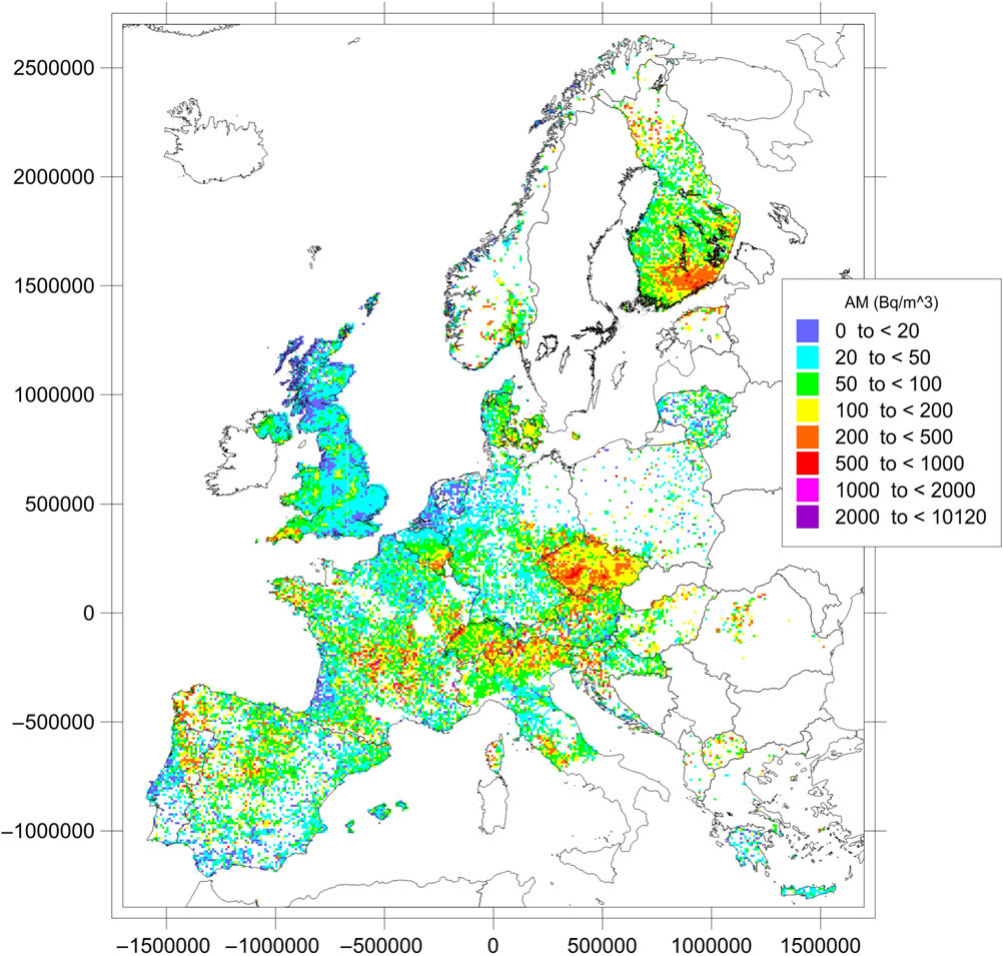
Arithmetic means over 10 × 10 km cells of long-term radon concentration in ground-floor rooms. Data available until May 2014 included.

Figure 2 shows the geographical distribution of cell means. Variations in indoor radon concentrations across Europe essentially reflect the underlying geology. Regions of high radon concentrations are found in the granitic areas of the Bohemian Massif, the Iberian Peninsula, the Massif Central, the Fennoscandian shield, Corsica, Cornwall and the Vosges Mountains, in the crystalline rocks of the Central Alps and karst rocks of the Swiss Jura and the Dinarides, the black shales in North Estonia and in certain volcanic structures in central Italy.

Table 1 gives descriptive statistics of the underlying dataset. The number of measurements per cell ranges from 1 up to nearly 24 000 (for a cell in the UK). The median number of measurements per cell is 4, with a median absolute deviation (MAD) of 3. While the AM for all cells in Europe (for all participating countries) is 98 Bq m^−3^, the median is 63 Bq m^−3^. Note, however, that this mean over cell means is not the mean exposure proxy but an estimate of the spatial mean (neither is it the mean of individual data nor the mean over country means).

**Table 1.** Descriptive statistics for the dataset on which the EIRM is based, as of May 2014.

Number of non-empty cells	21 340
Total number of measurements	823 394
Measurements per cell, MED±MAD	4±3
Min/Max number of measurements per cell	1/23 993
Cell mean, AM±CV %	97.6 Bq m^−3^±149
Cell median, MED±MAD	63.2±32.7 Bq m^−3^
Percentage cell AM > 300 Bq m^−3^	4.24 %
Percentage cell AM > 100 Bq m^−3^	30.1 %
CV within cells, MED±MAD	60.6±23.4 %
GSD within cells, MED±MAD	1.86±0.40

CV, coefficient of variation; CV, SD/AM; MAD, MED(|*x*-MED(*x*)|).

**Figure 3.**
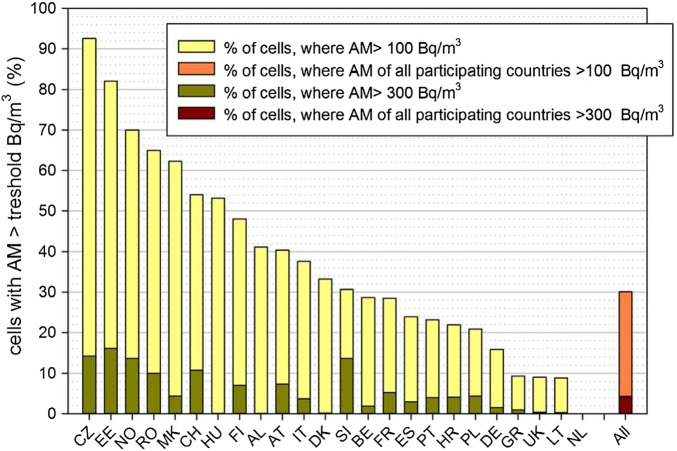
Percentage of cells with AM radon concentration of >100 and 300 Bq m^−3^ per country and for all participating countries.

Figure 3 shows the percentage of cells in which reference values of 100 and 300 Bq m^−3^ are exceeded. These thresholds have been motivated by proposals given by the WHO Handbook of Indoor Radon^(9)^ and the new European Basic Safety Standards^(10)^. For all participating countries, >30 % of the non-empty cells have an AM of >100 Bq m^−3^ and 4.2 % above 300 Bq m^−3^ (see Table 1). In the Czech Republic, >90 % of the AMs of all non-empty cells exceed 100 Bq m^−3^. The high percentage for Estonia must be interpreted against a sampling scheme targeted towards the high-radon areas in the north (see above). At the other end of the scale, none of the cells in the Netherlands have an AM above this level. This demonstrates how differently the countries are affected by the radon problem.

## THE EUROPEAN GEOGENIC RADON MAP

Because indoor radon concentrations are known to depend strongly on natural (geological) and anthropogenic factors such as construction types, building material and living habits and are temporally variable and characteristic for each house, the JRC has undertaken to map a variable that measures ‘what earth delivers’ in terms of radon potential (RP). This variable, ideally independent from anthropogenic factors and temporally constant over a geological timescale, is called the geogenic RP.

At a radon workshop held in Oslo in 2008, participants decided to create a European Geogenic Radon Map (EGRM), as a next step after the indoor map. While technical details remained open at the time, an expert group with a dozen members was formed. This group has since met at several workshops organised by the JRC and has gradually grown in number.

The idea to create an EGRM is also closely related to radon-prone areas, a concept which (mainly for semantic reasons) has been reformulated in the new European Basic Safety Standards^(10)^ as follows: ‘areas where the radon concentration (as annual average) in a significant number of buildings is expected to exceed the relevant national reference level’. Since a radon action plan is mandatory for all EU Member States under this Directive, the EGRM may support the Member States in implementing it.

It soon appeared that to generate a harmonised EGRM poses far greater conceptual and technical challenges than the indoor map. To discuss the concepts behind it is beyond the scope of this paper; the reader is referred elsewhere for details^(11, 12)^.

A first, trial version of such a geogenic map has been produced, using only geological units as mapping units. Each geological unit is assigned to a class, determined by its position in RP scale. Four RP classes, which may be termed low, moderate, elevated and high, were proposed. The default RP of the geological units is based on numerical RP data from Germany, the Czech Republic and Belgium. Defining the units is the underlying geology legend, based on the freely available geological map of the OneGeology project^(13)^.

When this trial map was first presented at the 11th International Workshop on the Geological Aspects of Radon Risk Mapping held in Prague in 2012^(14)^, some experts found the approach to be too simplistic and that geogenic radon classification needs to take into account more parameters. As other studies have shown^(15)^, there may be a large variability of RP within certain geological units, and a second division according to the geographical region may nearly be necessary to obtain geological units with a reasonable degree of uniformity for the radon risk.

Therefore, the JRC created a more comprehensive geogenic radon database and asked the participating countries to fill it with statistics of radiometric data related to radon. The intention is to collect as much information for as many geological units as found in Europe as possible, based on geological units linked to OneGeology. Where data are insufficient to classify the radon risk, it will be necessary to develop and apply transfer models. The idea is that the database should help to identify geologically similar units in other regions or countries where no measurement data exist, so that they can be used as default values. From this, the authors intend to construct a harmonised European map of geogenic radon, which will gradually evolve as more data or transfer models become available for geological units.

## THE EUROPEAN ATLAS OF NATURAL RADIATION

The European Atlas of Natural Radiation should be a collection of maps of Europe displaying the levels of natural radioactivity caused by different sources (indoor and outdoor radon, cosmic radiation, terrestrial gamma radiation and water). The overall goal of the Atlas is to estimate the annual dose that the public may receive from natural radioactivity, combining all the information from the different maps^(2)^.

While work will continue on the EIRM and EGRM, the next step for the Atlas will be to develop a cosmic radiation map and a terrestrial gamma radiation map. For this latter, the plan is to extract the natural terrestrial component from the total gamma dose data available from the EURDEP system^(16)^ (∼6000 stations) by subtracting cosmic, inherent background, artificial, airborne and deposited contributions.

To perform outdoor radon mapping, the idea is to use outdoor radon measurements available from European countries. Since the availability of such measurements may be limited, the authors are carrying out an interdisciplinary study of the correlation between meteorological, total gamma dose rate and outdoor radon concentration data in order to extract also this latter parameter from data available in the EURDEP system.

## CONCLUSIONS

Since it was started 8 y ago, the EIRM (as of May 2014) displays data from 24 countries on a 10 × 10 km grid across Europe. More than 21 000 cells have been filled with data, stemming from >800 000 individual measurements. Still, large variations occur in sampling density and coverage of territory between the participating countries and between regions within those countries. The number of measurements per cell ranges from 1 up to a maximum of ∼24 000. In total, the median number of measurements per cell equals 4, with an MAD of 3. Several countries have covered >100 % of their territory with data, due to a border effect. At the other end of the scale, a few countries have sampled (or sent data for) <20 % of their territory.

The indoor radon concentrations vary strongly within most countries, essentially reflecting the underlying geology. The AM of all non-empty cells in Europe (for all participating countries) is 98 Bq m^−3^, with a median of 62 Bq m^−3^.

Although the EIRM is slowly filling up, it is far from complete. Some countries (e.g. Ireland) had survey strategies, which produced datasets incompatible with the European grid, and others (e.g. Sweden) do not have a national database. The JRC will continue to collect data from new participants and from established ones as they complement or improve their data and will publish updated versions of the map from time to time.

In parallel to this effort, the EGRM aims to map ‘what earth delivers’ in terms of RP hazard, independent of anthropogenic factors. A first, trial map has been published and a database established to collect all available data relevant to the RP. Using this database, different approaches (multivariate classification, continuous) can be followed to create a harmonised European map.

In the longer term, a European Atlas of Natural Radiation will include other maps, such as cosmic rays, terrestrial gamma radiation and radiation from building materials. The plan is to combine these maps and calculate total exposure and dose caused by natural sources and their respective contributions in spatial resolution.
